# Supplementary material for: Effect of Konjac Glucomannan (KGM) on the Reconstitution of the Dermal Environment against UVB-Induced Condition
Source: Nutrients. 2020 Sep 11;12(9):2779. doi: 10.3390/nu12092779 (PMC7551622; doi:10.3390/nu12092779)
Supplement: Supplementary file 1 [file nutrients-12-02779-s001.pdf]

[Supplementary Information]

Effect of Konjac Glucomannan (KGM) on the Reconstitution of the Dermal Environment Against UVB-induced Condition

Kyung Ho Choi, Sung Tae Kim, Bum Ho Bin, Phil June Park

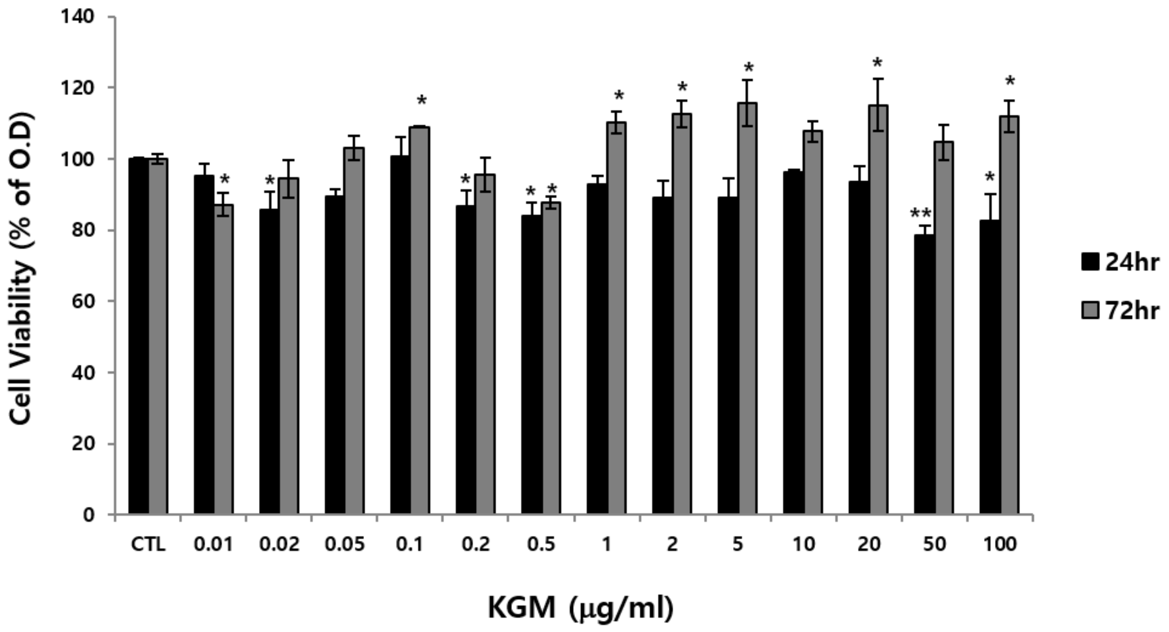

Supplementary Figure S1. The KGM has no significant effect on cell viability. Cell viability was measured in normal HEMns ($n = 3$) after treatment of various concentration 0.01-100 μg/ml of KGM for 24h and 72 h, respectively. Data are presented as means ± SD (*$p < 0.05$, **$p < 0.01$; unpaired Student's t-test).

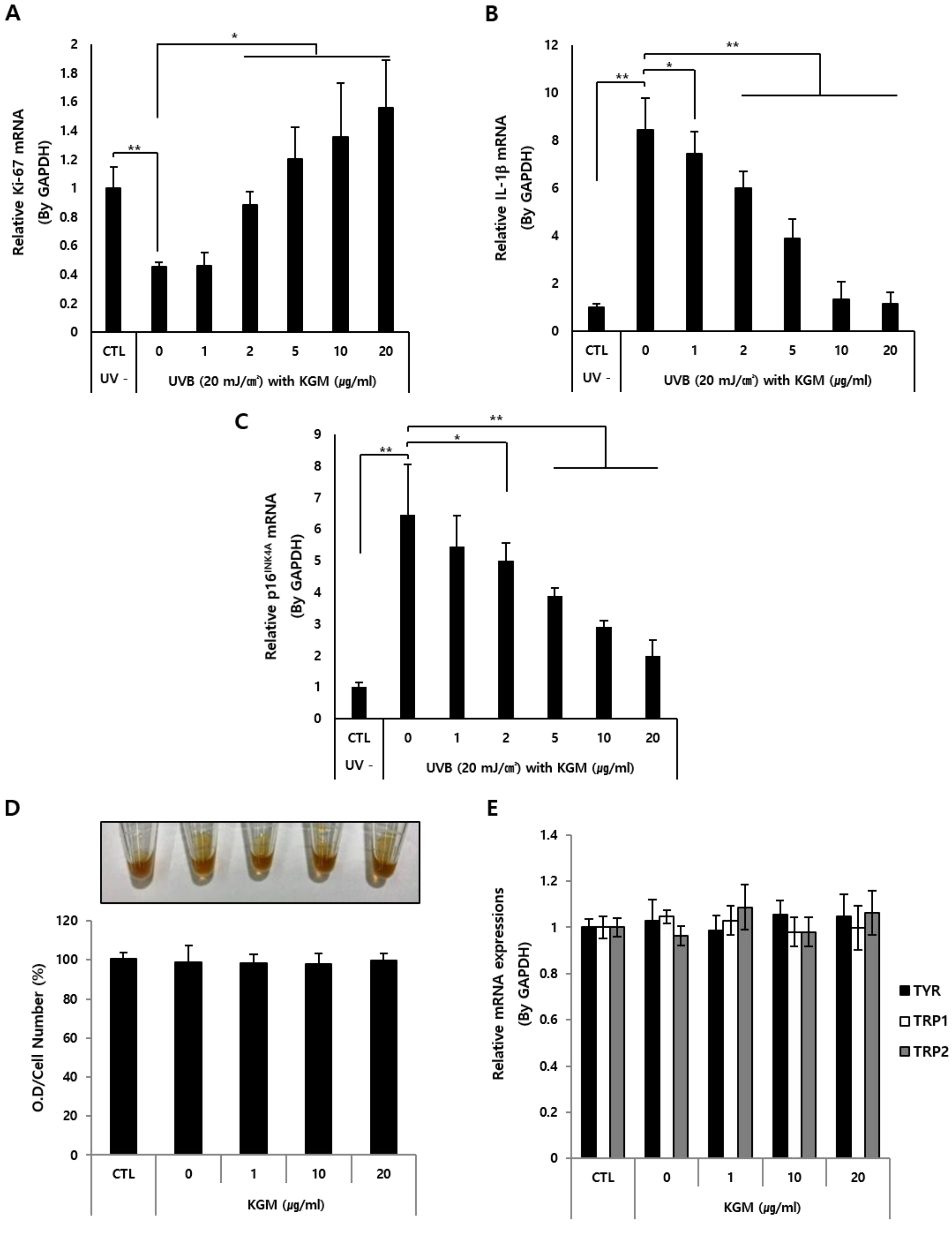

Supplementary Figure S2. The mRNA expression levels of cell growth, senescence were investigated in UVB-induced senescent HEMns and melanogenesis-related factors were investigated in normal HEMns after KGM treatment. (A) mRNA expression levels determined by RT-qPCR using specific Taqman probes for Ki-67, (B) IL-1β, and (C) p16^{INK4A}, respectively. Data are

presented as means ± SD (n = 3, *p < 0.05, **p < 0.01; unpaired Student's t-test). (D) The normal HEMns (n = 3) was cultured with various concentration of KGM for 14 days and the melanin contents were measured by absorbance at 450 nm. Each group were normalized by total cell count numbers. (E) The mRNA expression levels were determined by RT-qPCR using specific Taqman probes, such as TYR, TRP1 and TRP2. Data are presented as means ± SD (n = 3, *p < 0.05, **p < 0.01; unpaired Student's t-test).
